# Petrosamine Revisited. Experimental and Computational Investigation of Solvatochromism, Tautomerism and Free Energy Landscapes of a Pyridoacridinium Quaternary Salt

**DOI:** 10.3390/md21080446

**Published:** 2023-08-11

**Authors:** Christopher J. Gartshore, Xiao Wang, Yongxuan Su, Tadeusz F. Molinski

**Affiliations:** 1 Department of Chemistry and Biochemistry, University of California, 9500 Gilman Drive MC3568, La Jolla, San Diego, CA 92093, USA; gartcj@gmail.com (C.J.G.); xiao.wang1@merck.com (X.W.); yxsu@ucsd.edu (Y.S.); 2 Skaggs School of Pharmacy and Pharmaceutical Sciences, University of California, 9500 Gilman Drive MC3568, La Jolla, San Diego, CA 92093, USA

**Keywords:** alkaloid, pyridoacridine, acetylcholine esterase, merocyanine, marine natural product, NMR, UV-vis spectroscopy

## Abstract

Petrosamine (**1**)—a colored pyridoacridine alkaloid from the Belizean sponge, *Petrosia* sp., that is also a potent inhibitor of acetylcholine esterase (AChE)—was investigated by spectroscopic and computational methods. Analysis of the petrosamine-free energy landscapes, p*K*_a_ and tautomerism, revealed an accurate electronic depiction of the molecular structure of **1** as the di-keto form, with a net charge of *q* = +1, rather than a dication (*q* = +2) under ambient conditions of isolation-purification. The pronounced solvatochromism (UV-vis) reported for **1**, and related analogs were investigated in detail and is best explained by charge delocalization and stabilization of the ground state (HOMO) of **1** rather than an equilibrium of competing tautomers. Refinement of the molecular structure **1** by QM methods complements published computational docking studies to define the contact points in the enzyme active site that may improve the design of new AChE inhibitors based on the pyridoacridine alkaloid molecular skeleton.

## 1. Introduction

**Figure 1:**
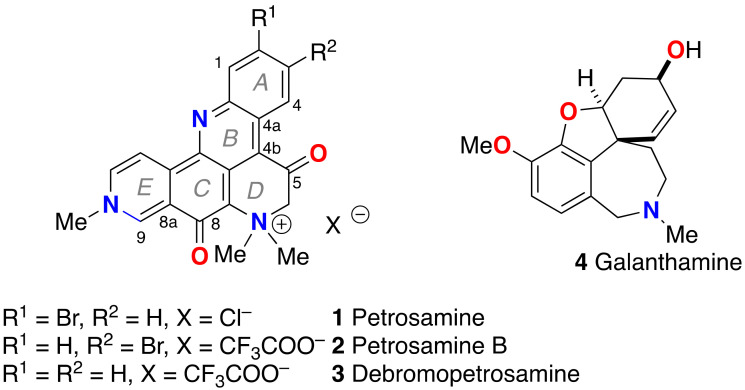
Structures of petrosamine (**1**), analogs **2**, **3** and galanthamine (**4**).

In 1988, Molinski and Faulkner reported the structure of petrosamine (**1**, Figure 1, a highly condensed pentacyclic alkaloid of intense color that was isolated from the sponge, *Petrosia* sp., collected at Carrie Bow Cay, Belize [1]. However, no bioactivity **1** was reported then, and a pronounced solvatochromism—the property of color changes (*λ*_max_) in solvents of different polarities—was observed. At the time, **1** represented the most complex structure in a growing class of pyridoacridine alkaloids from marine invertebrates [2]. Structurally, **1** can be considered a methylated and oxidized analog of the pentacyclic amphimedine—the first member of this class of alkaloids, described by Schmitz and co-workers in 1983 [3]. Unlike the structures of most pyridoacridines where the 3 nitrogen atoms are *sp*^2^ hybridized, **1** contains a quaternary ammonium salt: a *sp*^3^ quaternized N. Pyridoacridines manifest a range of biological activities including cytotoxicity, antineoplastic properties, antibacterial activity, and enzyme inhibition; a subject that has been extensively reviewed [4].

Since its initial report, alkaloid **1** has been reisolated from a Thai sponge species, *Petrosia* n sp. [5], and two new analogues have been described; petrosamine B (**2**) [6], a regioisomer of **1** from the Australian sponge *Oceanapia* sp. with modest inhibitory activity against aspartate semialdehyde dehydrogenase, and debromopetrosamine (**3**) from *Xestospongia* cf *carbonaria* collected in Palau [7]. Numerous marine natural products have been reported with neuroprotective properties in experimental models for neurodegenerative diseases, including acetylcholine esterase (AChE) inhibition [8]. Suwanborirux and coworkers showed that **1** is a potent inhibitor of AChE from the Pacific electric ray, *Torpedo californica* (IC_50_ = 91 nM) [5]; approximately six times more potent than galanthamine (**4**), an alkaloid used in the past for treatment of patients with Alzheimer’s disease (AD) to compensate neurotransmitter deficiency.

Preliminary docking studies of **1** with AChE revealed subtle electronic interactions with the putative receptor contacts [5]. Given the global rise of AD within aging populations and the therapeutic importance of AChE inhibitors for treatment, a detailed understanding of the electronic structure and molecular parameters for enzyme-inhibitor molecular contacts of **1** may advance design principles for new AD therapeutics. In this report, we present refined measurements of UV-vis, p*K*_a_ properties and quantum mechanical calculations of **1** that relate the observed solvatochromism through mapping of dipolar resonance forms to a simple model based on merocyanine dyes and stabilization of the ground state of the HOMO.

Petrosamine (**1**) exhibits several unusual physical and spectroscopic properties. The melting point of **1** is in excess of 300 °C suggesting high stabilization within the lattice energy of the crystalline form. Unlike most other pentacyclic pyridoacridines, the color of **1** is strikingly variable depending upon the physical state. Crystals of **1** are deep blue-purple but dilute solutions of **1** show highly complex UV-vis spectra due to long-wavelength absorption bands that manifest pronounced solvatochromism (changes in *λ*_max_ of **1** when measured in different solvents). For example, solutions of **1** in MeOH appear deep-blue (longest *λ*_max_ = 595 nm), but aqueous solutions of **1** appear purple *λ*_max_ = 574 nm) [1]. In dilute THF solutions (sparingly soluble) or DMSO, **1** appears green *λ*_max_ = 611 nm) [1]. In short, the range ∆*λ*_max_ of **1** is 36 nm in a range of solvents from H_2_O to DMSO. Petrosamine also displays pH indicator properties: addition of excess alkali to purple aqueous or blue MeOH solutions of **1**, changes the color to green, suggestive of a hyposochromic shift (∆*λ*_max_ < 0) mediated by the Brønsted acidity of the α-CH_2_ next to the keto group and deprotonation to the corresponding enolate.

The report by Quinn and coworkers describing the isolation of **2** by “fractionation on C18” and elution with a stepped gradient of acidic (CF_3_COOH) aqueous-MeOH [6] also reported solvatochromism in **2** very similar to that of **1**. Assignment of the structure of **2** was achieved through extensive analysis of ^1^H, ^13^C NMR and 2D NMR data (D_2_O-TFA-1% DMSO-*d*_6_). Compound **2** was depicted as a dication different from **1** in the resonance form of an O-protonated vinylogous amide embodying a fully aromatic quinolinium ring E [6]. Additional structural anomalies emerged. The assignment of C-8 in **2** is supported by an HMBC correlation from H-9 to C-8 (^3^*J*_CH_) based on the low ^13^C NMR chemical shift (*δ* 155.8 ppm), but no inter-ring HMBC correlations were reported for C-5 [6]. In contrast, the X-ray crystal structure of **1** [1] reveals two keto groups in the solid state (*d* (C-8–O) = 1.256 Å; *d* (C-5–O) = 1.203 Å), *d* (C-5–C-6) = 1.495(21). The C-O and C-C bond lengths are only compatible with a di-keto structure [9]. As the structures of **1** and **2** are very similar, consequently, the solution properties (p*K*a, UV-vis) should also be closely matched. Faulkner and Molinski reported the ^13^C NMR spectra (DMSO-*d*_6_) of **1** but not the assignments of the signals [1]. Suwanborirux and coworkers, reporting the ^13^C NMR spectrum of **1** in the latter solvent, assigned the signal at *δ* 161.3, s, the higher-field of the two most deshielded signals (*δ* 187.2, s; 161.3, s) to C-8 [5])

Comparisons of the NMR assignments of **1** and **2** are complicated further by measurements in different solvents and possible changes in tautomer or enolization states that accompany changes in pH and NMR solvent polarity, and H-bond donor-acceptor properties of the alkaloids. The latter factors affect the UV-vis properties of **1** and **2**. Surprisingly, neither the Quinn [6] nor Suwanborirux [5] groups claim to have observed the rapid enolization of **1** in D_2_O reported by Faulkner and Molinski that resulted in complete deuteriation of C-9 and ‘loss’ of the C-9 signal [1,10].

To reconcile these apparent paradoxes, it is required that a re-examination of **1** be given separate considerations of resonance in the native structure and possible tautomerism of **1** at the α-CH_2_ next to the C-5 C=O group. We undertook the task and completed additional solution spectroscopic measurements of **1** [NMR and electronic spectroscopy, UV-vis] and MS time-course studies and augmented by quantum mechanics calculations. The results presented here clarify several phenomena of **1**, including solvent dependence of HOMO-LUMO energies, kinetic and thermodynamic considerations of p*K*_a_, and tautomerism.

## 2. Results and Discussion

**Figure 2:**
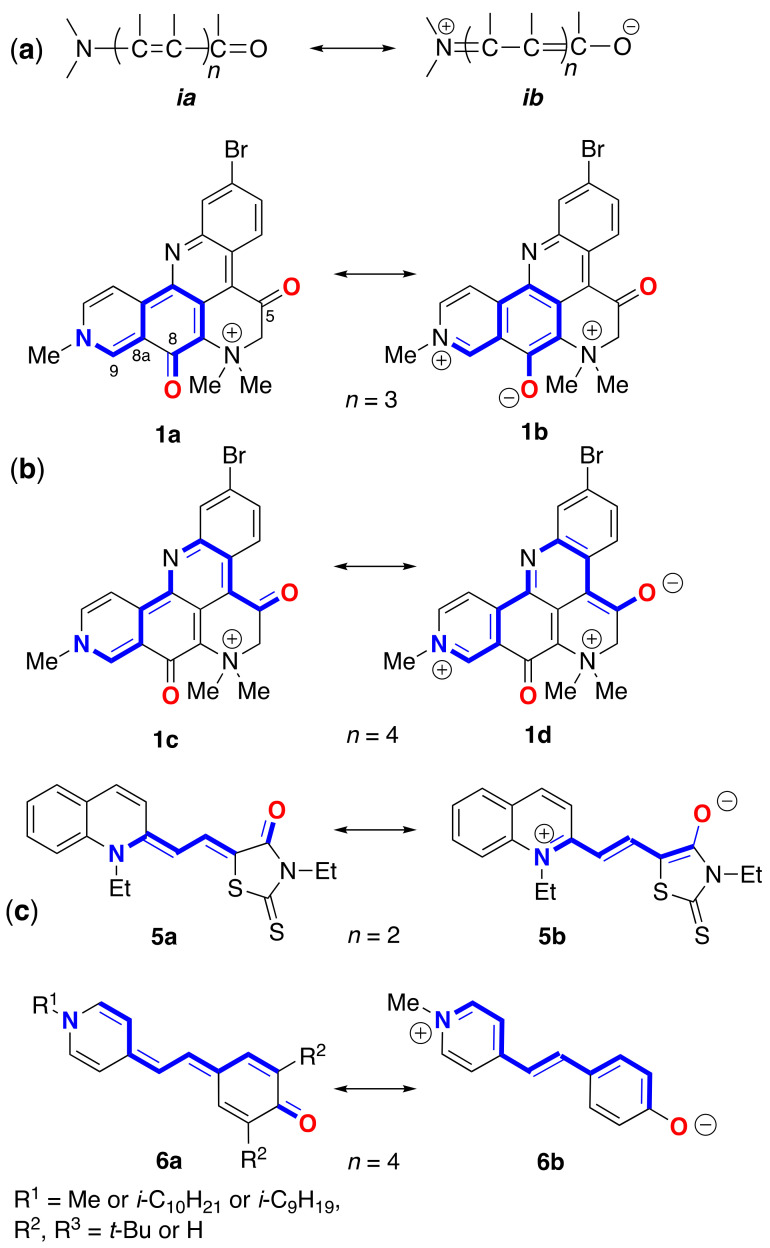
Canonical resonance forms—neutral (**a**) and dipolar (**b**)—for merocyanines defined by bond path, *n* (see [11]). (**b**) Petrosamine ‘neutral’ and dipolar resonance forms: **1a** (*n* = 3), **1b** (*n* = 2), **1c** (*n* = 4) and **1d** (*n* = 4). (**c**) Brooker merocyanine resonance forms; neutral (**5a**, **6a**) and dipolar (**5b**, **6b**) forms [11].

The solvatochromism of **1** and **2** is similar to lower-order merocyanine dyes, the canonical substructures of which can be discerned within the molecular framework of both natural products (Figure 2). For example, the same conjugated sub-structure in Brooker’s merocyanines [11,12] [generalized by Brooker as the vinylogous amide; neutral ***ia*** and ***ib*** dipolar (zwitterionic) resonance forms] is embedded in the molecular frameworks of **1** and **2**. Brooker merocyanines, for example, **5b** and **6b** (Figure 2), exhibit strongly positive solvatochromism (hypsochromism, or a blue shift in *λ*_max_ in polar solvents). It is not unreasonable to invoke the same spectroscopic and electronic properties of **5**—zwitterionic form with extended conjugation and stabilization of the ground state by solvation in polar solvents—as necessary and sufficient conditions to explain the solvatochromism of **1**–**3**.

While a complete description of the electronic properties of petrosamine (**1**) may be achieved in rigorous quantum mechanical calculations (see below), it is helpful for visualization purposes to consider Lewis’s bond formalism and resonance structures [13]. For clarity, only two pairs of resonance forms are depicted for **1** to illustrate the zwitterionic contributions of merocyanine substructures: the non-charged resonance forms **1a**,**c**, and ‘zwitterionic’ resonance form **1b**,**d**. Forms **1a**,**b** depict the shorter bond path (*n* = 3, c.f. ***ia***, ***ib***, Figure 2) and dipolar resonance forms **1c**,**d** show a longer bond path in an ‘aza-vinylogous amide’ (*n* = 4). The shortest bond path, *n* = 1 (not shown), would involve only the atoms C-8–C8a–C9–N-10), while the longest path, *n* = 4, evoking ‘particle-in-a-box’ formalism [14], best explains the long-wavelength UV-vis bands of **1** giving rise to its colors.

Solvatochromism of Brooker merocyanines has been rationalized [**11b**], using semi-quantitative valence resonance and frontier orbital theories, as arising from more extensive stabilization of the dipolar form **4a** of the ground state relative to the excited state in polar solvents. Accordingly, this differential stabilization increases the electronic transition energy, ∆*E*, due to a larger gap between the HOMO and LUMO of the longest wavelength transitions. The HOMO-LUMO gap is predicted to increase (blue-shifted absorption) in those structures with higher contributions from the zwitterionic resonance form **4b**, leading to more pronounced solvatochromism. More recent semi-empirical calculations (COSMO, PM3) of a different set of substituted Brooker merocyanines (**5**) by Morley and coworkers [15] supported stabilization of the ground state as mostly responsible for solvatochromism and predicted a larger role for hydrogen bonding in the zwitterionic form **5b** over the neutral form **5a**.

**Figure 3:**
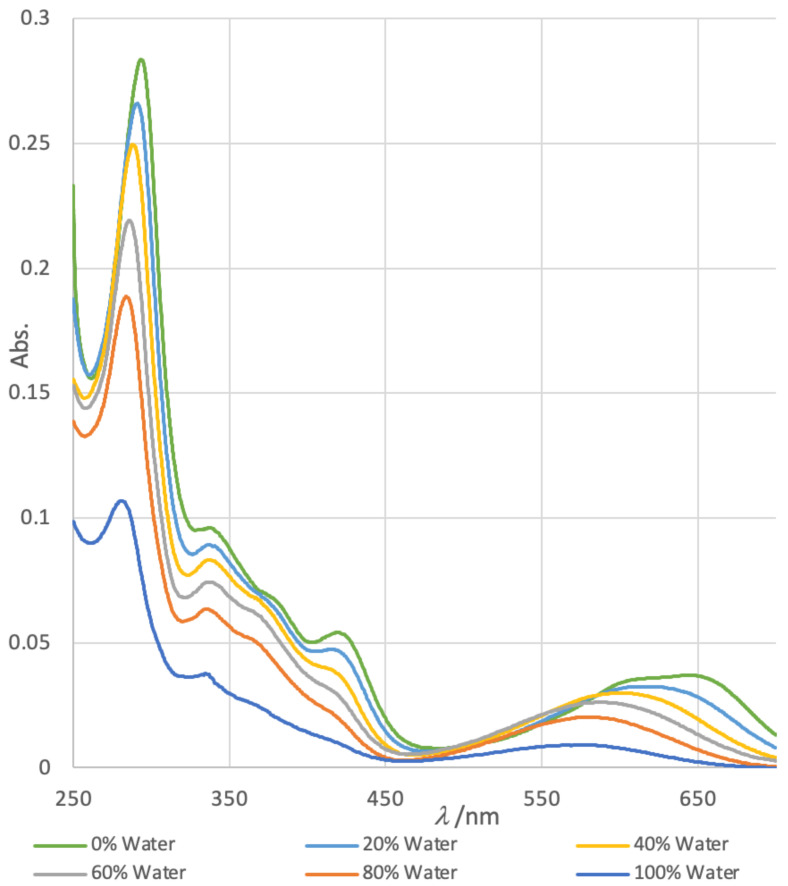
Solvatochromism in normalized electronic UV-vis spectra of petrosamine (**1**) in H_2_O-DMSO solvents of variable composition (H_2_O *v*/*v* = 0%, 20%, 40%, 60%, 80% and 100%).

The structure of petrosamine (**1**) appears to fulfil the criteria for merocyanine-type solvatochromism. Measurements of the UV-vis spectrum of solutions of **1** prepared in DMSO–H_2_O solvents of variable composition (Figure 3) exhibit changes in the *λ*_max_. Most prominently, the longest wavelength absorption band with the largest hypsochromic shift between 100% DMSO and 100% H_2_O (∆*λ*_max_ − 78 nm) is assigned to the forbidden *n*–π* transition that lends color to **1**, analogous to that of merocyanines.

A clear trend emerges band-1 (*λ*_max1_, defined here for convenience, as the dominant π-π* transition) shows a dramatic decrease in *ε* with increasing H_2_O content of the solvent and a weak bathochromic shift between 100% H_2_O to 100% DMSO (∆*λ*_max1_ 15 nm, Table 1). In contrast, the corresponding changes in band-2 include a strong blue shift (hypsochromism, *λ*_max2_ − 78 nm). The band *λ*_max2_ is affected most by solvents with increasing H_2_O content which parallels the reported behavior of Brooker merocyanines.

For comparison, we prepared the known merocyanine **6b** from 4-methylpyridine by the following sequence adapted from Minch and Sadiq Shah [16]: methylation (MeI, *i*PrOH, reflux), condensation of the resultant pyridinium methiodide with *p*-hydroxybenzaldehyde (piperidine, EtOH, reflux), and neutralization of the product **7** (*n*Bu_4_N^+^ HO^−^) to zwitterionic phenolate **6b**. Measurements of the UV-vis spectra of **6b** (*n*Bu_4_N^+^ salt) in mixed solvents (acetone-H_2_O, see Figures S3 and S4 in Supporting Information) showed a hypsochromic trend similar to **1** in DMSO-H_2_O. The long-wavelength (band-2) varied from *λ*_max_ (acetone) 591 nm to *λ*_max_ (H_2_O) 444 nm (∆*λ*_max_ − 69 nm) while *ε* changed only slightly across the range of solvent mixtures [17].

### 2.1. QM Calculations

**Figure 4:**
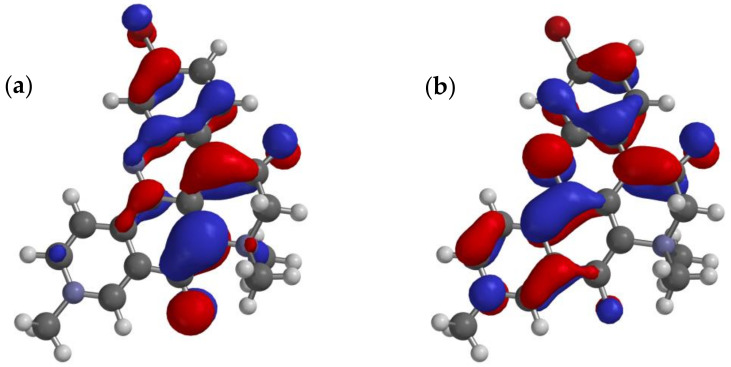
Calculated minimized structure and overlaid frontier π-orbitals of petrosamine (**1**) (DFT B3LYP 6-31G*, polarization continuum model = H_2_O) (**a**) HOMO and (**b**) LUMO.

The energies of electronic states of **1** were calculated using QM methods (DFT). Starting with the X-ray coordinates of **1**, the structure’s geometry was minimized using MMFF, then further refined by DFT (B3LYP 6-31G*, polarization continuum model = H_2_O). The 3D model of **1**, overlaid with frontier MOs, is shown in Figure 4. As expected, the HOMO-LUMO gap is relatively small (∆*E* = 2.4 eV). The orbital coefficients and calculated dipole moment of the excited state (*µ* = 19.2 D) are consistent with dominant contributions from the resonance form **1b** rather than **1a** and the strong donor properties of N-10. At first, this may seem anomalous, given the relatively low-field ^13^C NMR signal measured for C-5 in **1** (*δ* 187.4 [1] or 187.2 in **2** [5]); however, the additional deshielding effect is likely attributed to the inductive effect of the quaternized N-7.

**Figure 5:**
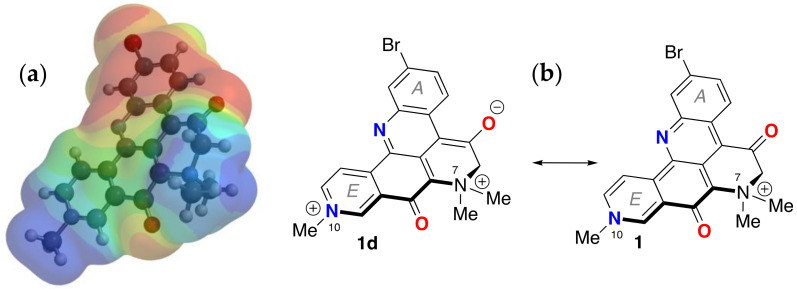
Minimized energy structure of petrosamine (**1**, DFT, Spartan’20, EDF2 6-31G, dipole moment *µ* = 19.2 D). (**a**) electrostatic potential surface and (**b**) the corresponding molecular framework of **1** (dipolar **1d** and ‘charge-minimal’ **1** resonance forms).

The electrostatic potential map of the minimized structure of **1** (Figure 5) clearly shows two loci of positive charge: one centered on the quaternized N-10, as expected, in ring D, and a second associated with the N atom in ring E. The latter supports charge-separated forms **1b**,**d** (Figure 2) in which N-10 participates as a donor group. Consequently, the formal bonding electron pair of N-10 is highly delocalized and can confer only weak basicity to **1**. Together with the UV-vis properties of **1**, the overall electron delocalization consolidates an electronic structure in which a zwitterionic partial structure strongly lends to charge separation, mostly in the ground state.

From an empirical viewpoint, the latter makes sense as the charge-separated forms **1b**,**d** preserve the aromaticity of rings A and E. A result of this delocalization is reduced basicity of N in ring E and, consequently, an expected lower p*K*_a_ for the conjugate Brønsted acid. Formally, adding H^+^ **1** to give a dication should sooner favor the C-8 or C-5 oxygen as an acceptor rather than N-10 or N-13. We find no evidence (NMR) for protonation of **1** at pH ~ 2, which attests to the overall poor basicity of **1**; an unsurprising finding given the permanent formal charge of +1 in this quaternary ammonium salt in all but the most basic or the most acidic media.

### 2.2. pK_a_ of Petrosamine (***1***), Does the Structure of ***1*** Exhibit Substantial Enol Content?

To estimate the p*K*_a_ of petrosamine, the UV-vis spectra of **1** were measured in buffered D_2_O at different pH. Across the pH 2–10 (Figure S1), the UV-vis spectrum of **1** is unchanged. From the Henderson-Hasselbach relationship (Equation (1)) [18] for Brønsted acid HA, the condition pH = p*K*_a_ is met when concentrations of conjugate species are equal ([HA] = [A^−^]). We surmise the p*K*_a_ of **1** lies outside this pH range (p*K*_a_ > 10). Indeed, a bathochromic shift in the UV-vis spectrum of **1** is only observed when a methanolic solution is treated with strong alkali NaOH (2 M, pH > 13). Conversely, only when a sample of **1** is dissolved in a very strong Brønsted acid (neat CF_3_COOH) are conditions met for a reasonable expectation of diprotonated petrosamine ([**1**•2H]^2+^). In the event when **1** was dissolved in neat TFA, the blue-purple color changed to bright yellow. The latter observation contrasts with the supposition drawn by Quinn and coworkers of doubly-protonated petrosamine B ([**2•**2H]^2+^) from their observation that **2** remains “*bright blue…when dissolved in methanol*”, under the relatively benign conditions used in C_18_-reversed phase isolation of the alkaloid (5% TFA-MeOH) [6]. In other words, N-10 in the monocationic molecules **1**–**3** is ‘pyridinium-like’ and non-basic (resembling **1d**), and the C-8 C=O is insufficiently Brønsted-basic to be substantially protonated under ordinary isolation conditions.

pH = p*K*_a_ + log_10_ [A^−^]/[HA] (1)

It is evident from the ^1^H and ^13^C NMR (DMSO-*d*_6_) that C-5 in petrosamine (**1**) is in the C8 keto form, a conclusion also reached by both the Suwanborirux and coworkers [5], and independently by Quinn and coworkers for petrosamine B (**2**) [6]. The single crystal X-ray crystallography of **1** is concordant with the C8 keto form. Solid state **1** is best represented by the di-keto tautomer, e.g., interatomic distances C8-O2, 1.256(17) Å; C5-O1, 1.203(15), and C5-C6, 1.495(21) [1].

### 2.3. Kinetic Measurements of Hydrogen-Deuterium Exchange of ***1***

**Figure 6:**
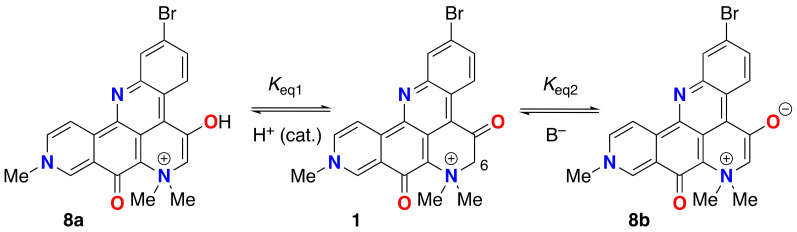
Keto-enol tautomerism of **1**. Enol **8a** (acid-catalyzed) and enolate **8b** (base-promoted).

None of the resonance forms of **1a**–**d** (Figure 2)—or more precisely, pathways of electron delocalization—explain the complete exchange of the H-6 protons by deuterium when **1** was dissolved in deuteric solvents (D_2_O, CD_3_OD) [1]. The latter can only be rationalized by consideration of the possibility of enolization (Figure 6), either through the positively charged **8a** in neutral to weakly acidic pH or the charge-neutral (zwitterionic) enolate **8b** under highly basic pH. The kinetic parameters for successive H replacement by D in **1**, defined by rate constants *k*_1_ and *k*_2_, are intrinsic properties as opposed to thermodynamic properties that relate to the equilibrium constants *K*_eq1_ and *K*_eq2_; the latter are largely dependent upon the strength and concentration of added base, B^−^ (Figure 6).

In hydroxylic solvents, **1** also resides largely in the di-keto form. However, we found that in both D_2_O and CD_3_OD, the C-6 methylene protons undergo rapid exchange to give the C-6 CD_2_-isotopomer (**1***-d*_2_) [1]; a rate so fast that we were unable to detect the C-6 CH_2_ or the intermediate forms within the time frame between sample preparation and measurement of the ^1^H NMR spectra. This observation was supported by ^1^H NMR, HSQC and HMBC measurements of **1** in protic solvent (CD_3_OH) where the C-6-CH_2_ group is still observable (HSQC correlation: H-6 to C-6, *δ*_H_ 4.64, s; *δ*_C_ 70.4 ppm). The simplest explanation for both phenomena is catalytic H-D exchange α-to the C-5 C=O group favored by an intermediate, the extensively-conjugated enol tautomer **8a** (Figure 6).

**Figure 7:**
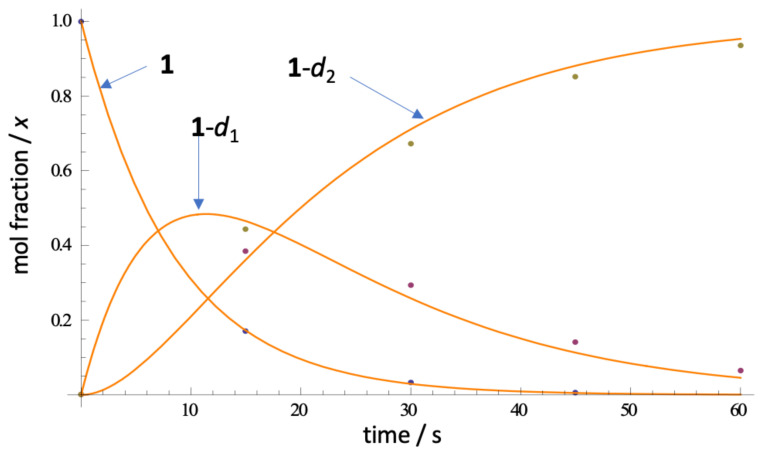
Representative ESIMS measurements of the rate of H-D exchange of petrosamine (**1** in CD_3_OD, 23 °C) and fitted curves (non-linear regression). See S6–S10 for rate law and *k* determinations.

To examine the kinetics of H-D exchange in **1** and place an upper bound on the rate of H-D exchange, the time-dependent appearance of the CD_2_-isotopmer by ESI mass spectrometry upon rapid dissolution of **1** in CD_3_OD (Figure 7). Under controlled conditions (23 °C, in situ measurement of the ESIMS of petrosamine with rapid sampling (sampled every *t* = 15 s) measurement—see Supplementary Materials (S6–S9 and Experimental (S14)), **1**-*d*_0_ (C_21_H_17_^89^BrN_3_O_2_, calculated *m/z* 422.0499 [M^+^]) quickly disappeared and was replaced by an ephemeral isotopologue **1**-*d*_1_ (*m*/*z* 423.0561), followed by convergence upon **1**-*d*_2_ (*m*/*z* 424.0624). Complete exchange (>90%) was observed within 90 s of dissolution of **1**. From triplicate measurements and rapid sampling, we could fit the kinetic deuterium exchange data to a first-order rate law. We estimated the apparent first and second H-D exchange rate constants to be (Table 2), *k*_1_ = 0.131(68) s^−1^ and *k*_2_ = 0.0755(26) s^−1^, respectively (best R^2^; see S6–S10). As expected, rate constant *k*_2_ is about half of *k*_1_, consistent with the expected rate law (*k*_1_/*k*_2_ = 1.74(94)), negating the involvement of a substantial primary kinetic isotope effect.

The exchange of **1** to **1**-*d*_2_, in the absence of added acid, appears to be much faster than H-D exchange rates of other phenones, e.g., propiophenone (p*K*_a_ 24.4, DMSO [19] α-tetralone (p*K*_a_ 24.7 [20,21]. The rates of acid-promoted keto-enol equilibration of acetophenone (p*K*_a_ 18.4 ± 0.03) have been measured. For example, the rate of ketonization of acetophenone enol (1-phenylethen-1-ol) is linearly dependent upon [H_3_O^+^] with a catalytic efficiency determined to be *k*_H+_ = (1.25 ± 0.02) × 10^3^ M^−1^s^−1^ [22]. We conclude that tautomerism of **1**, too, must be very fast, even in the absence of added acid. Either the enol **8a** or enolate **8b**, although undetectable as a discrete species in the time frame of ^1^H NMR, present a pathway to the acid-base equilibria of **1** and **1**-*d*_2_, but enolization of the keto form is likely dominant.

Some amount of discussion has been given on the keto-enol state of **1**. The NMR data for **1** reported by Suwanborirux and coworkers support the C5 keto form in DMSO-*d*_6_ [5] and—in agreement with Molinski and Faulkner, the enol form in D_2_O or CD_3_OD [1]—but Quinn and coworkers find, “*no evidence for this keto-enol isomerism*” in **2**. Likely, the *K*_eq_ of keto-enol tautomerism lies on some continuum, dependent upon solvent dielectric and H-bond donor ability.

We find it unlikely that the position of the Br in ring *A*—the only difference between structures **1** and **2**—would exert a profound difference in physicochemical behavior. Quinn’s argument—“*no involvement for this keto-enol isomerism*” in **2**—is confounded by two uncertainties: the composition of their NMR solvent (TFA, D_2_O, DMSO-*d*_6_) is not specified quantitatively, and facile interpretation of the ^13^C NMR signals for the C8 signal: “*in contrast C-8 resonated at 155.8 ppm, supporting it as a phenolic resonance. It was, therefore, more likely that petrosamine also exists in the C-8 enol form and C-8* [Molinski and Faulkner [1] *was the carbon at 161 ppm*”, contrasts with those of Suwanborirux (*δ* 174.7 ppm, DMSO-*d*_6_) and their observation of, “*the broad methylene carbon at δ_C_ 69.2*” (*DMSO-d*_6_, “*100 µmol NaOD*” [5]). For comparative purposes, we synthesized a model quinoline **S1** (see Supplementary Material)—a merocyanine of class (*n* = 3, see labeling of **1a**,**b**) and found the ^13^C NMR chemical shift of C8 in CD_3_OD to be *δ*_C_ 172.2 ppm. Aside from these points, none of the three different ^13^C NMR values for C8 in **1** and **2** are incompatible with the structures, both of which are not strictly aryl ketones but vinylogous amides, the dominant resonance form of which will be highly dependent upon solvent dielectric and H-bond donor properties.

The observed ^1^H and ^13^C NMR spectra of petrosamine (**1**), and the C6 exchange to CD_2_ in CD_3_OD/NaOD [5], support the enol form in hydroxylic solvents. It is likely fast exchange between the hybrid structure of **1** and **8a**,**b** with an equilibrium constant *K*_eq1_ largely in favor of **1** in DMSO-*d*_6_, but moving to the dominant form **8a** in hydroxylic solvents. The enolate **8b** may be favored as the catalytically important intermediate under ‘neutral’ conditions for reasons of charge neutralization, but insufficient data preclude testing this hypothesis with more certainty. In either case—enol or enolate intermediate—we surmise the electron-withdrawing quaternized N^+^Me_2_ group in **1** and the related petrosamines, **2, 3**, plays a significant role in accelerating the rates of H-D exchange and lowering the p*K*_a_ of the C-6 CH_2_ group.

**Figure 8:**
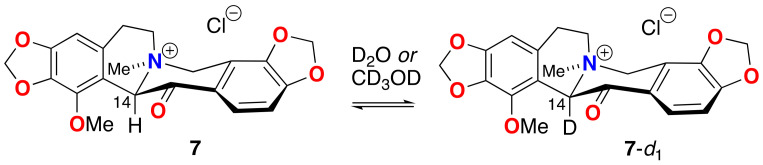
Rapid H-D exchange of coulterberbinone (**7**) in deuteric solvent under ambient conditions.

To the best of our knowledge, rapid H-D exchange of a substituted β-quaternary ammonium ketone within a natural product has been observed only in one other instance, coulteroberbinone (**7**, Figure 8 [23]), an *N*-quaternary ammonium isoquinoline alkaloid from the leaves of *Romneya trichocalyx*. The authors note the C-14 C-H in **7**, assigned to the α-proton between the carbonyl and quaternized nitrogen (*δ*_H_ 5.64, s), underwent rapid proton-deuterium exchange in D_2_O or CD_3_OD to C-D (**7**-*d*_1_) under ambient conditions (supported by ^1^H NMR and ESIMS data).

Two major factors most likely explain the relatively low p*K*_a_ of **1** and **7**: the electron-withdrawing (inductive) effect of the -N^+^Me_2_ quaternary ammonium group (N-7) and stabilization of enol **5** (or enolate **6**) through extensive conjugation of the heteroaromatic core, not unlike the stabilization of the enolates of alkyl phenones, e.g., acetophenone, determined by UV-vis (p*K*_a_ = 18.4). Indirect determinations of the p*K*_a_ of the enol of acetophenone have also been obtained from the kinetics of reactions of acetophenone: e.g., α-chlorination [24] and aminolysis of the corresponding enol acetate [25]. In contrast, **1** appears to undergo rapid enolization without added Brønsted acid, suggesting that this tautomerization may even be autocatalytic.

Is the enol **8a** of petrosamine (**1**) present in substantial concentrations? From the ^1^H NMR spectrum of **1** (DMSO-*d*_6_), we detect no signals attributable to **8a**. It can be ascertained from ^1^H NMR that *K*_eq1_ (Figure 7) is very small (estimated from the limits of integration, *K*_eq1_ < 0.05), and the equilibrium of tautomers lies well towards the keto form **1**. As noted above, in highly-basic aqueous solutions of **1**, the charge-minimal enolate **8b** appears in substantial concentrations, placing a lower boundary of p*K*_a_ ~ 15 for **1**. In the absence of base, the mechanism of H-D exchange **1** to **1**-*d*_2_ in CD_3_OD most likely engages substantial equilibrium concentrations **8a** to allow rapid exchange of the CH_2_ to CD_2_ in deuteric solvents within less than 2 min at 23 °C. An accurate p*K*_A_ of **1** is not accessible from measurements in aqueous solvents, and securing an estimate will likely require measures in a suitable aprotic solvent (e.g., titration in DMSO [20,26]). Bordwell values of p*K*_a_ are conventionally obtained by titration of a solution of the weak Brønsted acid in DMSO with its non-nucleophilic conjugate base, ‘dimsyl sodium’ (CH_3_(S=O)CH_2_^−^ Na^+^) with Ph_3_CH as an indicator [26] where a colored endpoint is presented by the deep-red Ph_3_C^−^ anion. In the case of **1**, the high color of the substrate and its conjugate base should lend itself to ‘self-indicating’.

## 3. Materials and Methods

### 3.1. General Experimental Procedures

Inverse detected 2D NMR spectra were measured on a Jeol ECA (500 MHz) spectrometer equipped with a 5 mm ^1^H{^13^C} 5 mm probe or a Bruker Avance III (600 MHz) NMR spectrometer with a 1.7 mm ^1^H{^13^C} microcryoprobe. ^13^C NMR spectra were collected on a Varian NMR spectrometer (125 MHz) with a 5 mm Xsens ^13^C{^1^H} cryoprobe. NMR spectra were referenced to residual solvent signals, (CD_3_)_2_CO (*δ*_H_ 2.05, *δ*_C_ 29.8). High-resolution ESITOF analyses were conducted on an Agilent 1200 HPLC coupled to an Agilent 6230 TOFMS. Low-resolution MS measurements were made on a Thermoelectron Surveyor UHPLC coupled to an MSD single-quadrupole detector. HPLC was performed on an Agilent 1200 HPLC. UV-vis spectra were measured on a Jasco V-630 spectrometer in quartz cells (1.00 cm path length, Helma). FTIR spectra were collected on thin film samples using a Jasco FTIR-4100 fitted with an ATR accessory (ZnSe plate). Optical densities (OD, *λ* nm) in microplate wells were measured using a Molecular Devices Spectramax 384 Plus. Measurements of pH were made with a digital pH meter (Denver Instrument, Arvada, CO, USA, Model 220), calibrated against standard solutions (NaH_2_PO_4_-Na_2_HPO_4_).

### 3.2. UV-Vis Measurements

Standard solutions of accurately-weighed **1** and **6b** were prepared in volumetric flasks and used for serial dilutions to the final working concentrations in specified media, either pure HPLC grade solvent (DCM, DMSO, acetone or DMF—see Supporting Information for **6b** in acetone) or mixtures of aqueous HPLC solvents of defined H_2_O composition.

*pH Dependence*: Britton-Robinson buffer was prepared by dissolving 2.48 g of boric acid, 2.30 mL acetic acid and 2.72 mL phosphoric acid in 1 L of water and titrating in a 2.0 M solution of NaOH to the desired pH.

40 μL of a 2.37 mM of petrosamine (**1**) in MeOH was added to 960 μL of the desired buffer solution, and the UV-vis spectrum was measured from *λ* 190–700 nm.

*Solvent-Dependence*: Solutions of **1** in H_2_O (95.0 μM) and acetone (95.0 μM) were mixed in the desired ratio, and the UV-vis spectrum was measured from *λ* 320–700 nm.

### 3.3. DFT Calculations of ***1***

The X-ray coordinates of petrosamine (**1**) were used as a starting point for DFT calculations of the energy, geometry minimized structure, polar isosurfaces and LUMO-HOMO. Calculations were performed within the Spartan ’20 package [27]. DFT energy minimized structure of **1** was obtained using B3LYP with the 6-31G* basis set under a polarization continuum model (=H_2_O). Calculated minimized structure with overlaid frontier π-orbitals and electrostatic potential isosurfaces of petrosamine (**1**) are shown in Figure 4 and Figure 5.

### 3.4. Synthesis of Merocyanine Dye (***6b***) [16]

Iodomethane (3.11 mL, 50 mmol) was slowly added to a cold mixture of 4-methylpyridine (4.86 mL, 50 mmol) and 2-propanol (5 mL). The stirred mixture was heated at reflux overnight. Removal of the solvent gave the crude 4-methylpyridinium methiodide (10.23 g) as an off-white solid. A portion of the latter salt (3.00 g, 12.8 mmol), 4-hydroxybenzaldehyde and piperidine (1.06 mL, 10.7 mmol) were dissolved in anhydrous EtOH (16 mL) and heated at reflux overnight, with stirring. Upon cooling, a red precipitate was deposited. The red solid was filtered (Büchner funnel), dissolved in KOH solution (0.2 M, 17 mL, 15 mmol), and heated with stirring for 30 min. Blue-red shiny crystals were recovered by filtration, washed with cold water, and dried to give **6b** (2.00 g, 65%). The ^1^H NMR of the sample was consistent with the expected product.

### 3.5. H-D Exchange Measurements of ***1*** by ESI-TOFMS

H-D exchange measurements were made by ESI-TOFMS using the following conditions: VCap: 3500V; fragmentor voltage: 160 V; nozzle voltage: 500 V; drying gas temperature: 325 C, sheath gas temperature: 325 C, drying gas flow rate: 7.0 L/Min; sheath gas flow rate: 10 L/min; nebulizer pressure: 40 psi. A petrosamine (**1**) sample was rapidly dissolved in CD_3_OD (23 °C). An aliquot of the solution was immediately introduced into the inlet of the TOF mass spectrometer, and the ratios of **1**-*d*_0_, **1**-*d*_1_ and **1**-*d*_2_ were measured ‘on the fly’ from the corresponding molecular ion intensities [M]^+^ normalized to the [M]^+^ of a solution of **1** in CH_3_OH at the same concentration. Subsequent measurements were made at 15 s intervals over a total reaction time of 90 s. HRMS data were acquired and stored in centroided mode. See Figure 7 and S6–S9.

## 4. Conclusions

Evidence supports that the major tautomer of petrosamine is the C5 and C8 di-keto form **1**, both in solution and solid states. While the enol form **8a** was not detectable by ^1^H NMR in DMSO-*d*_6_, it is nevertheless likely responsible for the H-D exchange of **1** in deuteric solvents. The exceptional solvatochromism of **1** is best attributable to charge delocalized resonance forms **1a** and **1d**—partial structures within **1** that are analogous to Brooker merocyanine dyes [11]. As with the latter, the hyposochromic solvent effects mostly correlate with polar-solvent stabilization of the ground state (HOMO) and attribution to the color changes of **1**. The complete isotopic exchange of the C6 CH_2_ group in **1** to CD_2_ in a deuterated solvent under ambient conditions is supported by ^1^H NMR and fast-sampled ESIMS measurements. The exchange mechanism likely proceeds through rapid acid-catalyzed keto-enol tautomerism.

Refinement of the molecular structure of **1** by QM methods maps the electron delocalization and accompanying charge distribution in **1**. These and other refinements complement computational docking studies that can lend a more precise definition of host-guest interactions of **1** in its cognate enzyme active site. In turn, these observations may assist in the design and synthesis of new AChE inhibitors based on the pyridoacridine skeleton: a privileged alkaloid molecular framework produced exclusively by marine invertebrates.

## Figures and Tables

**Table 1:** UV-vis properties of *λ*_max1_ ^1^ and *λ*_max2_ ^1^ in petrosamine (**1**) in DMSO-H_2_O ^1^.

% H_2_O (*v/v*)	*λ*_max1_ (nm)	*ε*_1_ ^1^	*λ*_max2_ (nm)	*ε*_2_ ^1^
0	296	184,000	648	24,000
20	290	172,000	622	20,600
40	288	161,000	604	19,400
60	287	141,700	589	16,500
80	282	122,252	581	12,900
100	281	68,500	570	4700

^1^ See text for definitions. *ε*_1,2_ values normalized from the literature value of *λ*_max2_ (*ε* 4700) [1].

**Table 2:** Rate constants *k*_1_ and *k_2_* for H-D exchange in **1** in CD_3_OD (23 °C, see Figure 7) ^1^.

Reaction	*k*_1_ (s^−1^)	*k*_2_ (s^−1^)	95% Confidence Interval
**1** –> **1**-*d*_1_	0.1311(68)	–	{0.1176, 0.1447}
**1**-*d*_1_ –> **1**-*d*_2_	–	0.0755(26)	{0.0702, 0.0807}

^1^ See S6–S10 for plotted raw ESIMS data and detailed kinetic analysis.

## Data Availability

Original data will be made available upon reasonable request.

## Supplementary Materials

The following supporting information can be downloaded at: https://www.mdpi.com/article/10.3390/md21080446/s1, Figure S1: UV-vis spectra of **1**. pH dependence in Britton-Robinson buffer (normalized); Figure S2: FTIR of **6b** (film, ATR); Figure S3: UV-vis spectra of **6b** in pure acetone; Figure S4: UV-vis spectra of **6b** in acetone-H_2_O mixtures (normalized); Figure S5: In situ ESIMS measurements of the rate of H-D exchange of **1** in CD_3_OD; Figure S6: Derivation of kinetic parameters of H-D exchange of **1** in CD_3_OD and uncertainty analysis; Figure S7: HRMS. Time course of **1** in CD_3_OD (triplicate runs) and Fitted Curves; Figure S8: ESI-TOFMS of **1** collected in (a) in CH3OH (b) CD_3_OD at 30 s) (c) CD_3_OD at 90 s; Figure S9: Time course of ESI-TOF-MS for petrosamine (**1**); Figure S10: ^1^H NMR of **1** in a. CD_3_OH and b. CD_3_OD after 5 min; Figure S11: ^1^H NMR temperature study of petrosamine (**1**) (CD_3_OH, 500 MHz); Table S1: *λ*_max_ and absorbance of merocyanine dye **6b** in H_2_O-acetone mixtures. Table S2: ^1^H and ^13^C NMR of **1** in CD_3_OD and CD_3_OH; Table S3: ^1^H NMR data for compounds **S1** and **S6** (CD_3_OD, 500 MHz); Scheme S1: Synthesis of compound **S1**: Experimental: Synthesis of Model Compound **S1**. References [28,29] are cited in the Supplementary Materials.
