# Supplementary material for: Petrosamine Revisited. Experimental and Computational Investigation of Solvatochromism, Tautomerism and Free Energy Landscapes of a Pyridoacridinium Quaternary Salt
Source: Mar Drugs. 2023 Aug 11;21(8):446. doi: 10.3390/md21080446 (PMC10455967; doi:10.3390/md21080446)
Supplement: Supplementary file 1 [file marinedrugs-21-00446-s001.zip › marinedrugs-2530064-supplementary.pdf]

Supporting Information

Petrosamine Revisited. Experimental and Computational Investigation of Solvatochromism, Tautomerism and Free Energy Landscapes of a Pyridoacridinium Quaternary Salt.

Christopher J. Gartshore[1], Xiao Wang[1], Yongxuan Su[1] and Tadeusz F. Molinski[1,2,*]

1 Department of Chemistry and Biochemistry, University of California, San Diego, 9500 Gilman Drive, La Jolla, CA 92093, USA MC3568; gartcj@gmail.com

2 Skaggs School of Pharmacy and Pharmaceutical Sciences, University of California, San Diego, 9500 Gilman Drive MC3568, University of California, San Diego, 9500 Gilman Drive, La Jolla, CA 92093, USA.

* Correspondence: tmolinski@ucsd.edu

Page	Title	Content
S1.	**Figure S1**	UV-vis spectra of **1**. pH dependence in Britton-Robinson buffer (normalized).
S2	**Figure S2**.	FTIR of **6b** (film, ATR)
S2.	**Figure S3**.	UV-vis spectra of **6b** in pure acetone
S3.	**Figure S4.**	UV-vis spectra of **6b** in acetone-H_2O mixtures (normalized)
S3.	**Table S1.**	λ_{max} and absorbance of merocyanine dye **6b** in H_2O-acetone mixtures.
S4.	**Figure S5**.	In situ ESIMS measurements of the rate of H-D exchange of **1** in CD_3OD.
S5.	**Figure S6**	Derivation of kinetic parameters of H-D exchange of **1** in CD_3OD and uncertainty analysis.
S6	**Figure S7**	HRMS. Time course of **1** in CD_3OD (triplicate runs) and Fitted Curves.
S7-S9	**Figure S8**	ESI-TOFMS of **1** collected in (a) in CH_3OH (b) CD_3OD at 30s) (c) CD_3OD at 90s.
S10.	**Figure S9.**	Time course of ESI-TOF-MS for petrosamine (**1**).
S11.	**Table S2.**	^{1}H and ^{13}C NMR of **1** in CD_3OD and CD_3OH.
S11	**Figure S10**.	^{1}H NMR of **1** in **a**. CD_3OH and **b**. CD_3OD after 5 min.
S12.	**Figure S11.**	^{1}H NMR temperature study of petrosamine (**1**) (CD_3OH, 500 MHz).
S13.	**Scheme S1.**	Synthesis of compound **S1**.
S14.	**–**	Experimental: Synthesis of Model Compound **S1**.
S15.	**Table S3.**	^{1}H NMR data for compounds **S1** and **S6** (CD_3OD, 500 MHz).

Figure S1. UV-vis spectra of **1**. pH dependence in in Britton-Robinson buffer[1] (pH 2 – 10, normalized)

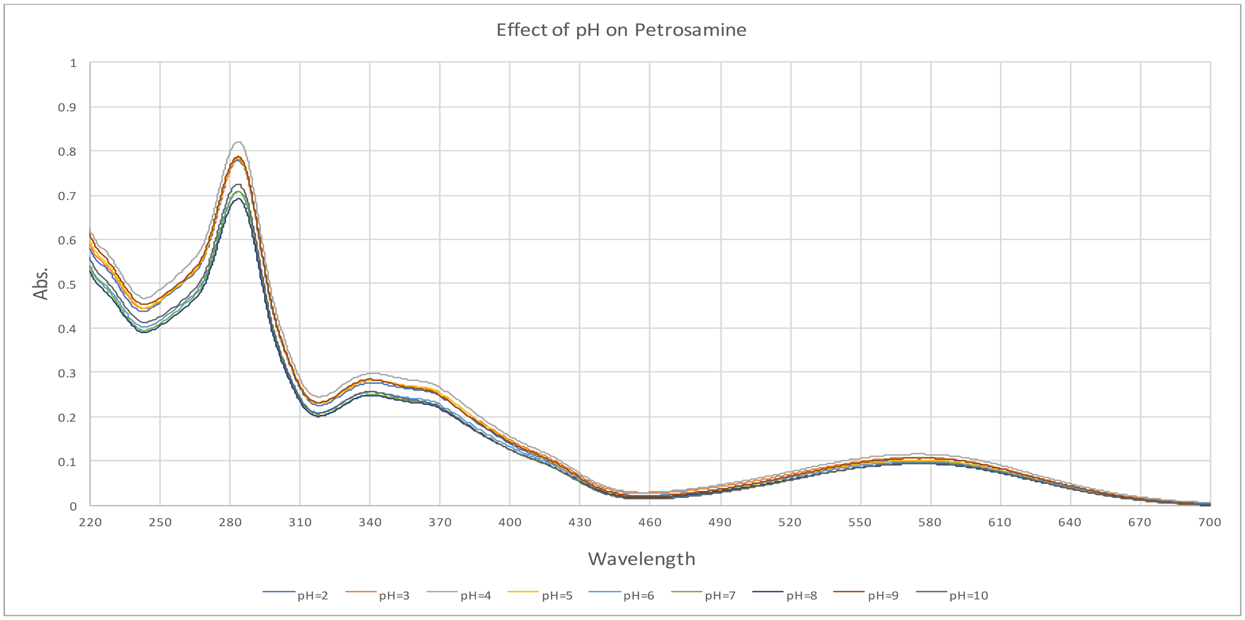

[1]Britton, H. T. K.; Robinson, R. A. CXCVIII.—Universal buffer solutions and the dissociation constant of veronal. *J. Chem. Soc.* **1931**, 1456-1462.

Figure S2. FTIR spectrum of **6b**

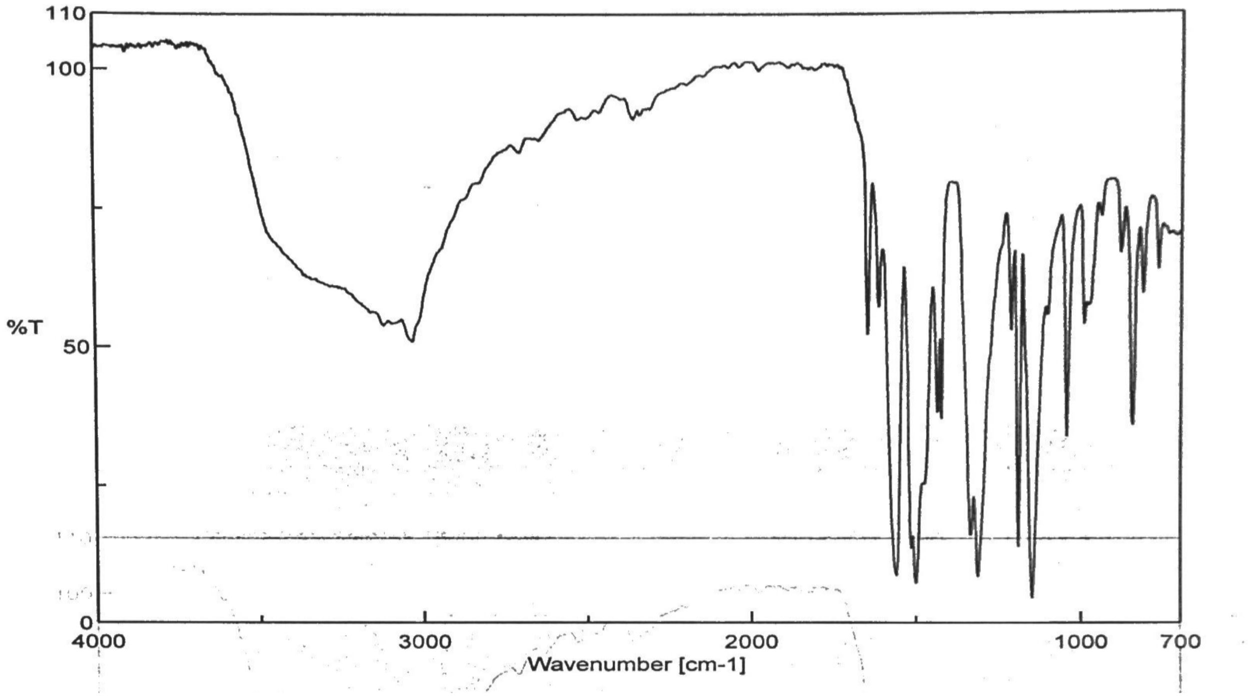

Figure S3. UV-vis spectrum of 6b in acetone (lmax2 = .

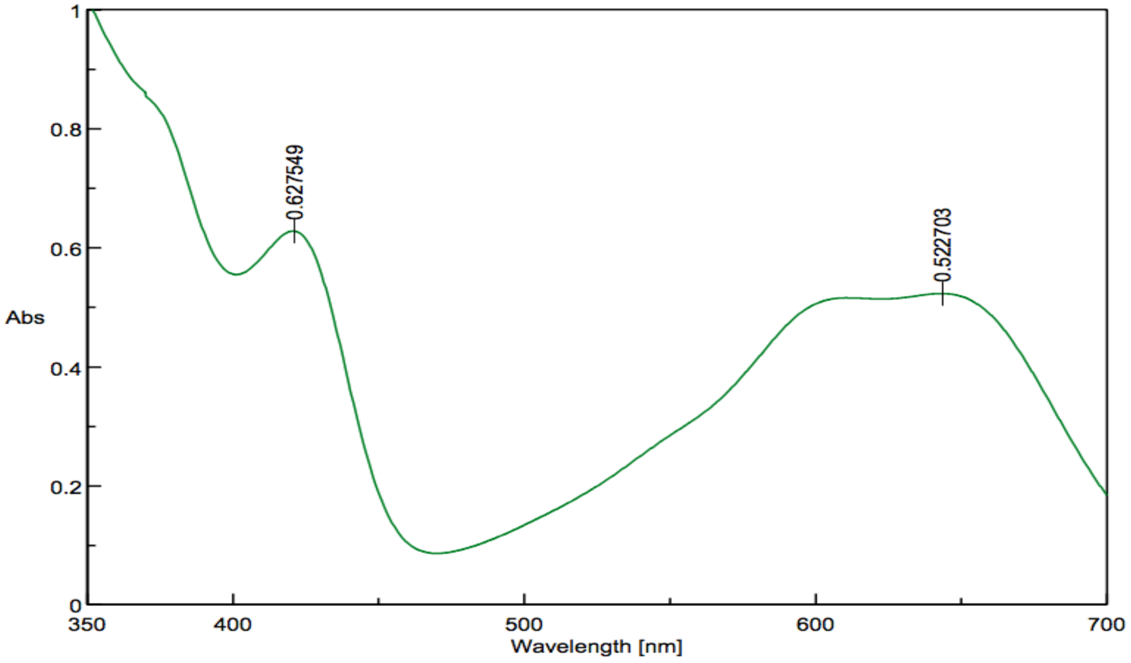

Figure S4. UV-vis spectra of **6b** in acetone-H_2O mixtures[1] (normalized).

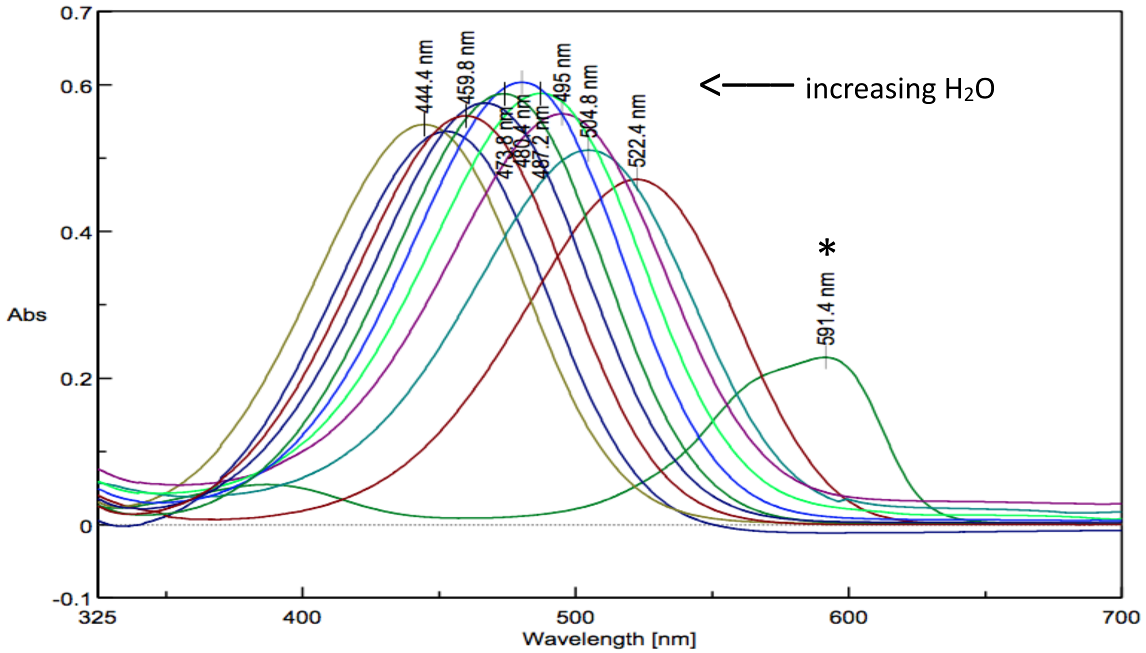

[1]Each with 5 μL $n\text{Bu}_4\text{N}^+$ HO^-: 0% H_2O*, 20%, 30%, 40%, 50%, 60%, 70%, 80%, 90% and 100%).

Table S1. λ_{max} and absorbance of merocyanine dye **6b** in H_2O-acetone mixtures.

%H_2O (v/v)	λ_{max2}[1]	Absorbance
0	591.4	0.227779
10	522.4	0.459048
20	504.8	0.510442
30	495.0	0.559982
40	487.2	0.587949
50	480.4	0.603025
60	473.8	0.587191
70	466.8	0.574550
80	459.8	0.557193
90	450.8	0.535364
100	444.4	0.545338

[1]See text for definition of 'band-1' and 'band-2'

Figure S5. In situ ESIMS measurements of the rate of H-D exchange of **1** in CD_3OD.[1]

[1]'t = 0' values normalized to a solution of **1** CH_3OH

Figure S6. Derivation of kinetic parameters of H-D exchange of petrosamine (**1**) in CD_3OD and uncertainty analysis.

Petrosamine (**1**) was dissolved in CD_3OD and aliquots of the solution sampled by ESIMS every 15 s from 15 s to 120 s. Three replicate runs were completed.

Assuming the kinetics of each of the two elemental steps in H-deuterium exchange reaction follow first-order rate laws and that the reverse reactions can be ignored (high concentration of CD_3OD), the following analysis is valid (note: A = **1**-d_0, B = **1**-d_1 and C = **1**-d_2,

$$A \xrightarrow{k_1} B \xrightarrow{k_2} C$$

The reaction rate of A, B and C could be defined by eq. (1)-(3), and their initial concentrations were shown in eq (4)

$$\begin{cases} \dfrac{d[A]}{dt} = -k_1[A] & (1) \\ \dfrac{d[B]}{dt} = k_1[A] - k_2[B] & (2) \\ \dfrac{d[C]}{dt} = k_2[B] & (3) \\ [A]_0 = 1,\ [B]_0 = [C]_0 = 0 & (4) \end{cases}$$

The solution to the system of the differential equations above were shown in eq (5)-(7) (also see *note*):

$$[A]_t = e^{-k_1 t} \qquad (5)$$

$$[B]_t = \frac{k_1(e^{-k_2 t} - e^{-k_1 t})}{k_1 - k_2} \qquad (6)$$

$$[C]_t = \frac{k_2 e^{-k_1 t} - k_1 e^{-k_2 t}}{k_1 - k_2} + 1 \qquad (7)$$

k_1 and k_2 were then obtained by fitting experimental [A], [B] and [C] from the three repetitions to eq (5), (6) and (7). Only the kinetic data of the first 90s were used for fitting.

	k (s^{-1})	Standard Error (σ)	95% Confidence Interval
k_1	0.1311	0.0068	{0.1176, 0.1447}
k_2	0.0755	0.0026	{0.0702, 0.0807}

Figure S7. HRMS. Time course of **1** in CD_3OD (triplicate runs) and Fitted Curves.

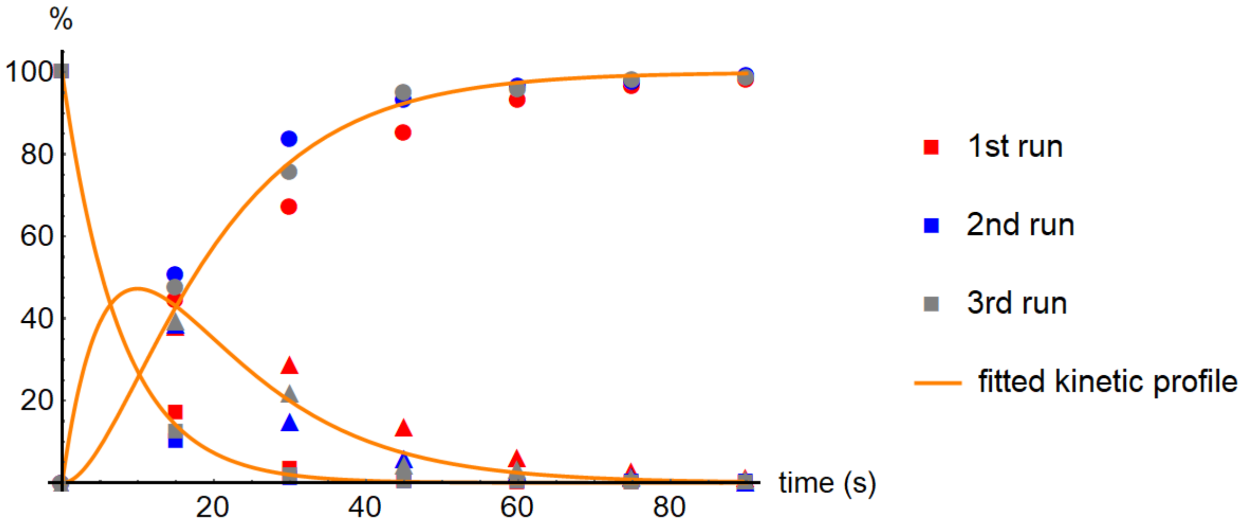

.

Figure S8: ESI-TOFMS of Petrosamine (**1**) collected in (**a**) in CH_3OH (**b**) CD_3OD at 30s (**c**) CD_3OD at 90s.

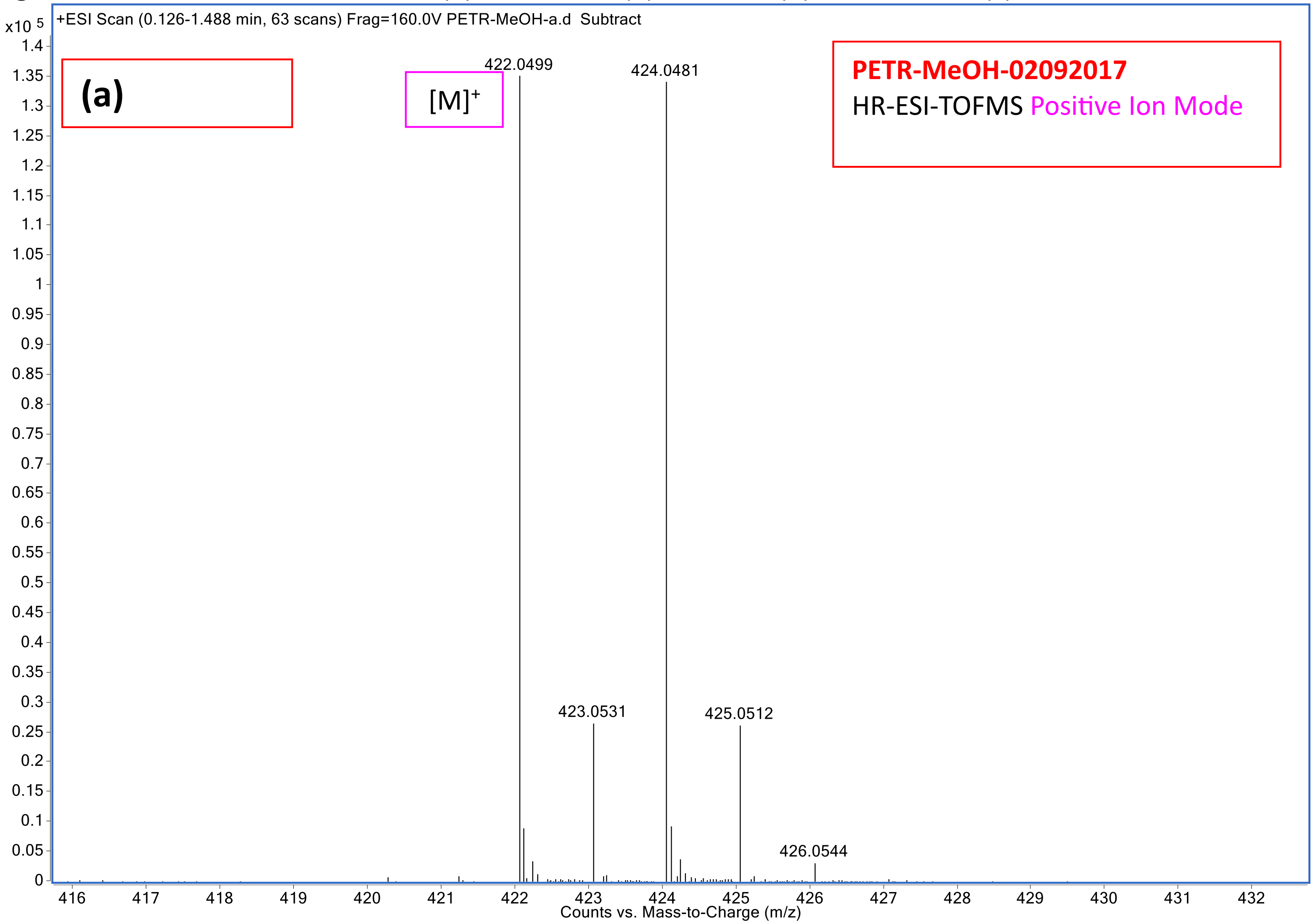

Search Results: Sample PETR-MEOH-02092017

Mass Measured	Theo. Mass	Delta (ppm)	Composition
422.0499	422.0499	0.0	$[C_{21}H_{17}BrN_3O_2]^+$

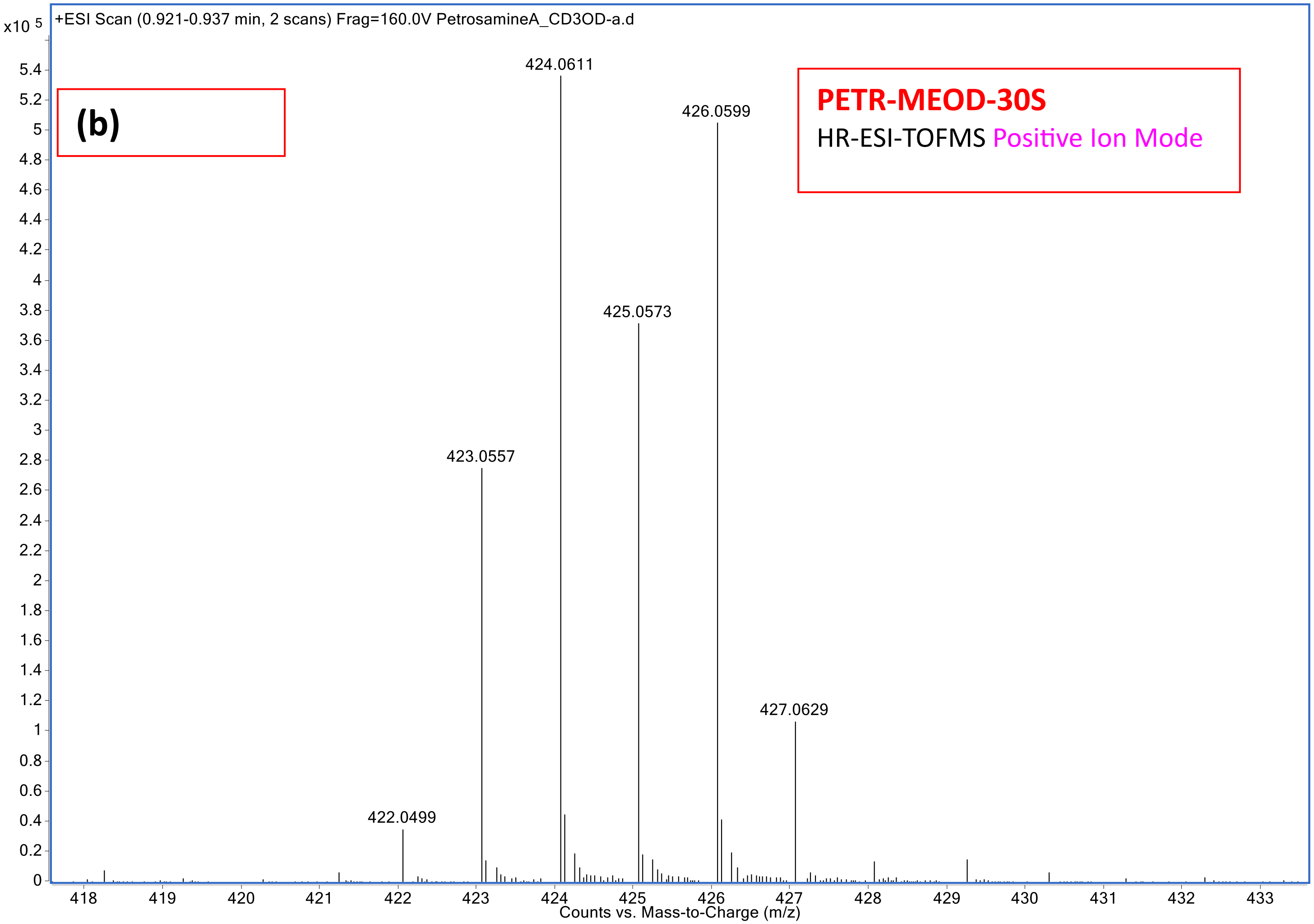

Search Results: Sample PETR-MEOD-30S

Mass Measured	Theo. Mass	Delta (ppm)	Composition
424.0625	424.0624	0.2	$[C_{21} H_{15} D_2 Br N_3 O_2]^+$

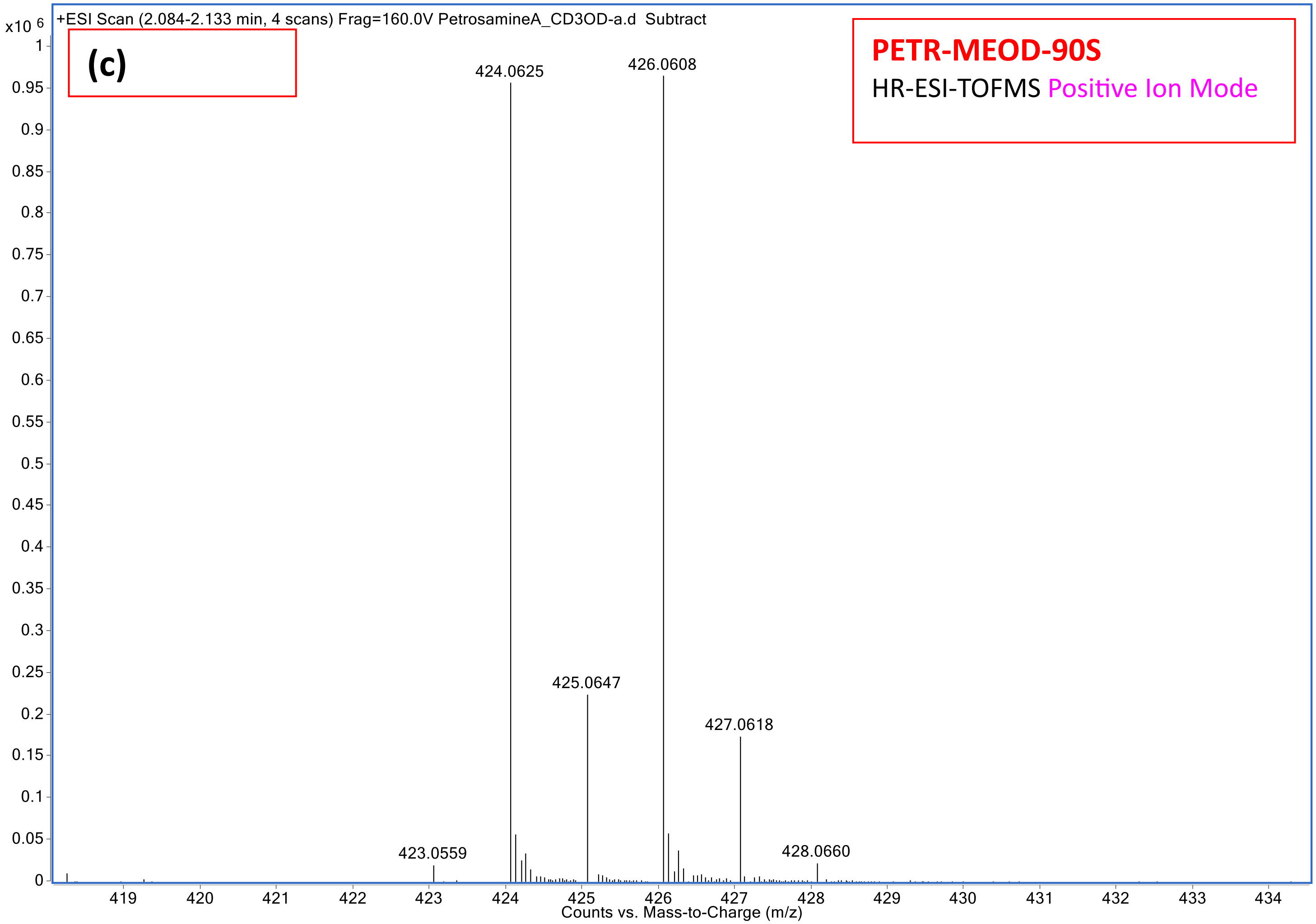

Search Results: Sample PETR-MEOD-90S

Mass Measured	Theo. Mass	Delta (ppm)	Composition
424.0625	424.0624	0.2	$[C_{21}H_{15}D_2BrN_3O_2]^+$

Figure S9. Time course of ESI-TOF-MS for petrosamine (**1**). Samples measured at 15 s intervals for 120 s.

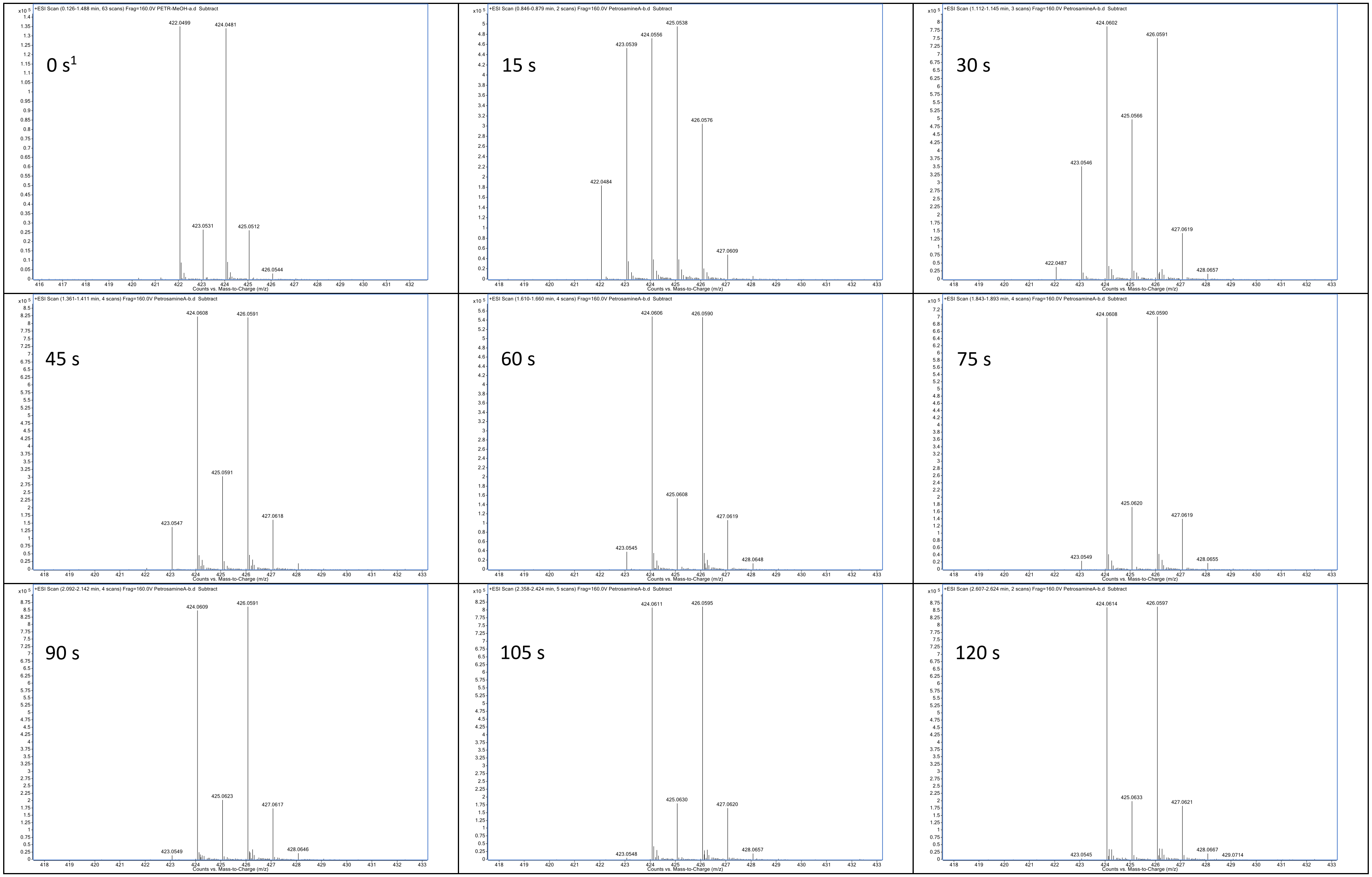

[1]Measurement in CH_3OH.

Table S2. ^{1}H and ^{13}C NMR of **1** in CD_3OD and CD_3OH (enol form, **8a**).

atom	^{13}C (CD_3OD)	^{1}H (CD_3OD)	^{1}H (CD_3OH)
1	135.5	8.41 (d, J = 1.8 Hz, 1H)	8.40 (d, J = 2.0 Hz, 1H)
2	122.5		
3	136.7	7.88 (dd, J = 1.8, 9.0 Hz, 1H)	7.87 (dd, J = 2.0, 9.0 Hz, 1H)
4	127.6	9.23 (d, J = 9.0 Hz, 1H)	9.22 (d, J = 9.0 Hz. 1H)
4a	124.5		
4b	117.1		
5	187.9		
6	71.2 (HMBC)	Missing	4.64 (s, 2H)
7-N(CH_3)$_2$	54.4	3.93 (s, 6H)	3.93 (s, 6H)
7a	116.2		
8	162.3		
8a	133.5		
9	146.4	9.80 (s, 1H)	9.80 (s, 1H)
10-NCH_3	49.2	4.66 (s, 3H)	4.66 (s, 3H)
11	143.0	9.06 (bs, 1H)	9.04 (dd, J = 1.0, 6.5 Hz, 1H)
12	123.7	9.50 (bs, 1H)	9.54 (d, J = 6.5 Hz, 1H)
12a	141.6		
12b	144.5		
12c	130.0		
13a	144.7		

Figure S10. ^{1}H NMR (expansion) of **1** in (**a**). CD_3OH and (**b**). CD_3OD after 5 min.

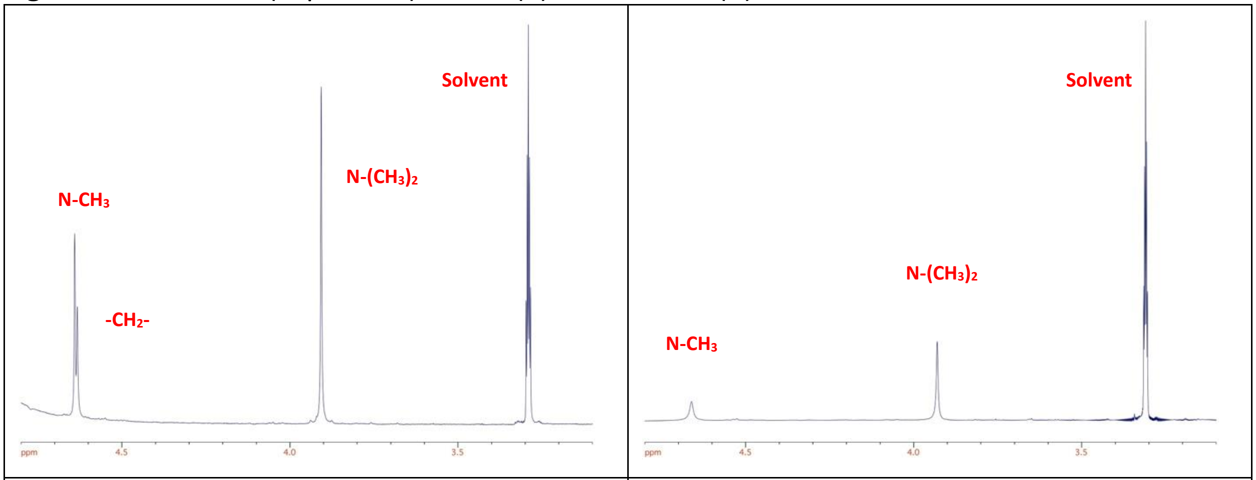

a. Petrosamine (**1**) in CD_3OH | b. Petrosamine (**1**) in CD_3OD (5 min.)

Figure S11. ^{1}H NMR temperature study of petrosamine (**1**) (CD_3OH, 500 MHz)

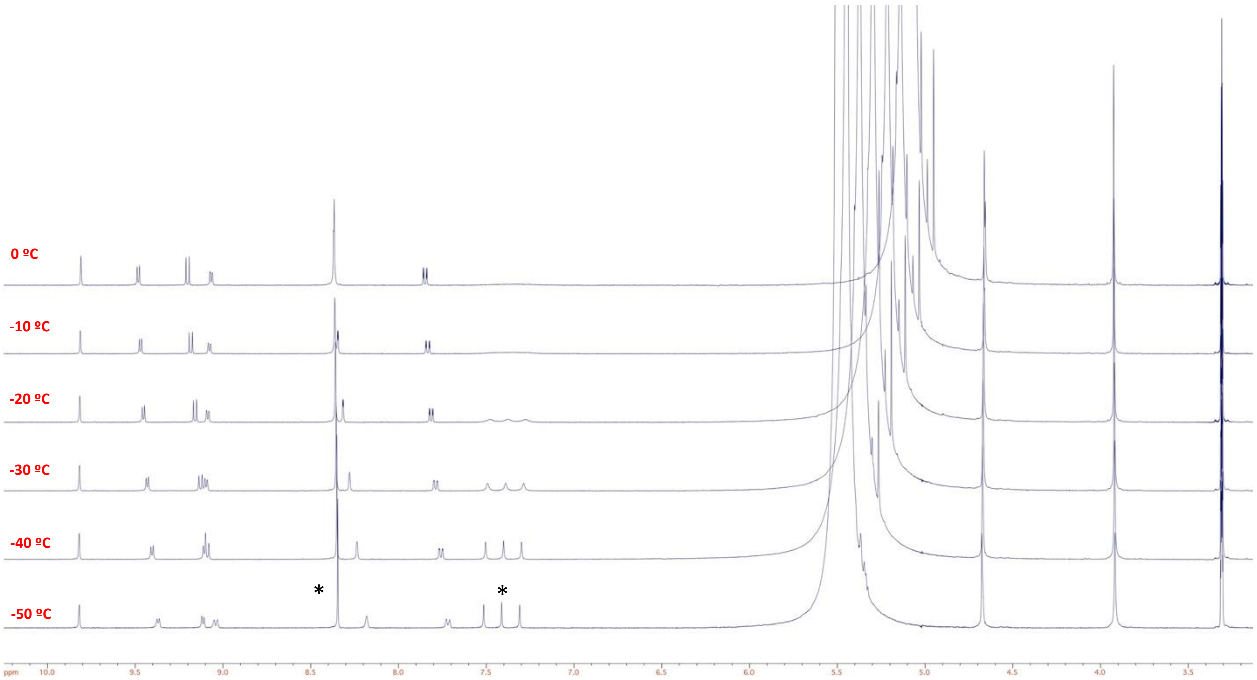

*Ammonium formate (NH_4^+ $HCOO^-$) from HPLC purification: δ 7.41 ppm (t, $^1J_{1H\text{-}14N}$ = 15.0 Hz) and δ 8.35 ppm.

Synthesis of **S1** *:* Compounds **S3** and **S4** were synthesized using literature methods.[2] Condensation of 2-methoxybenzaldehyde with aminoacetaldehyde dimethyl acetal **S3** gave a Schiff base which was treated with ethyl chloroformate and trimethylphosphite to give a carbamate-phosphonate intermediate which cyclized when treated with $TiCl_4$ to give 8-methoxyisoquinoline **(S4**, 38% yield). The latter was treated with BBr_3 to give 8-hydroxyisoquinoline **S5** which was heated with MeI in a sealed tube to afford the *N*-methylisoquinoline **S6**. Treatment of **S6** with K_2CO_3 afforded the desired zwitterionic **S1.**

Scheme S1. Synthesis of model zwitterion **S1**.[2]

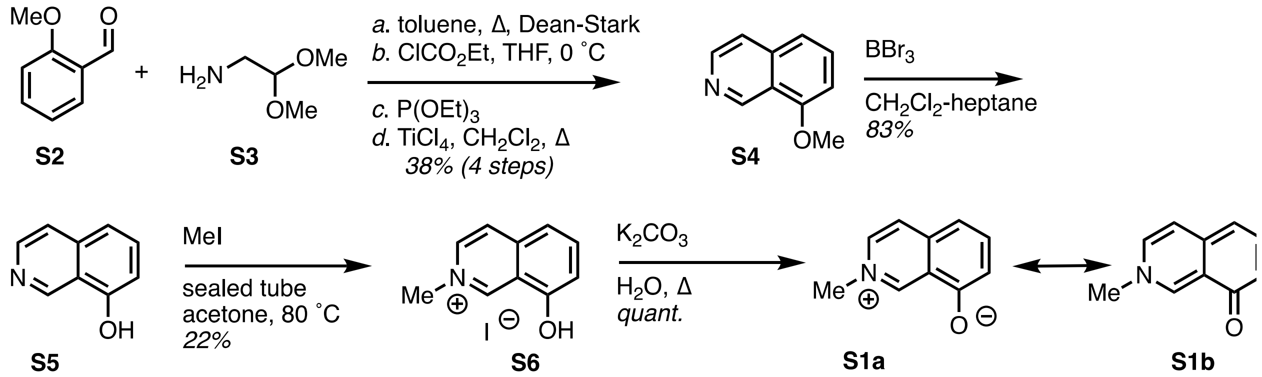

Comparison of the ^{13}C NMR data for compound **S6** and **S1** reveals that the C-7 resonance changes from δ 158.7 in the phenol form to δ 172.2 in the phenoxide form (Table 1). The corresponding carbon in petrosamine (C-8) resonates at δ 162 ppm. This is also supported by the proton signal for H-9, in the phenol form it appears at δ 9.85 ppm, but at δ 9.27 ppm in **S1** . Although depicted as zwitterionic **S1a,** the structure – like similar heterocyclic vinylogous amides – is likely better drawn as a quinolone (viz. **S1b**[3]). The corresponding proton, H9 in petrosamine (**1**) appears at δ 9.80 ppm. However, this theory is inconsistent with the NMR and MS data. If the phenol form was present then in DMSO-d_6 the exchangeable OH should be present in the ^1H NMR spectrum however it was not observed. Additionally, after a prolonged time in deuterated solvent (~ 1 week in CD_3OD) the –OH should exchange with deuterium giving an M+3 amu peak in the mass spectrum, which again was not observed.

---

[2] Graulich A.; Scuvée-Moreau, J.; Seutin, V.; Liégeois, J.-F. *J. Med. Chem.* **2005**, *48*, 4972 – 4982.
[3] Molinski, T.F.; Faulkner, D.J. *Tetrahedron Lett.* **1988**, *29*, 2137-2138.

Synthesis of Model Compound S1.

8-Hydroxy-2-methylisoquinolinium iodide **(S6).**

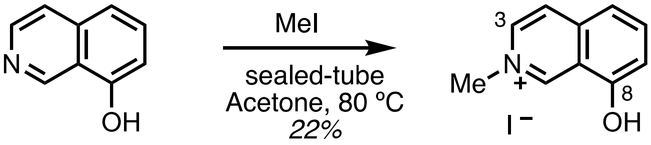

S5 **S6**

8-Hydroxyisoquinoline (**S5**) (140 mg, 0.96 mmol, 1.0 eq.) was dissolved in acetone (10 mL) in a sealed tube and MeI was added (0.3 mL, 4.82 mmol, 5.0 eq.) and the mixture heated at 80 ºC for 12 h. After this time, the reaction mixture was cooled, diluated with Et_2O (20 mL), and the resulting precipitate filtered off and dried under vacuum to give **S6** as a yellow solid (60 mg, 0.2 mmol, 22%). IR ν_{max} 3413, 1645, 1578, 1373, 1298, 1200 1106 cm^{-1}; ^1H NMR (CD_3OD, 500 MHz) δ 9.85 (s, 1H), 8.42 (d, J = 8.0 Hz, 1H), 8.28 (d, J = 8.0 Hz, 1H), 8.05 (t, J = 10.0 Hz, 1H), 7.66 (d, J = 10.0 Hz, 1H), 7.27 (d, J = 10.0 Hz, 1H), 4.49 (s, 3H); ^{13}C NMR (CD_3OD, 125 MHz) δ158.7, 147.7, 140.3, 139.2, 136.3, 126.3, 120.7, 118.4, 114.1, ~49.0 (under solvent); HRESITOFMS *m/z* 160.0754 $[M–I]^+$ (calcd. for $C_{10}H_{10}NO$, 160.0757).

2-Methylisoquinolin-2-ium-8-olate **(S1)**

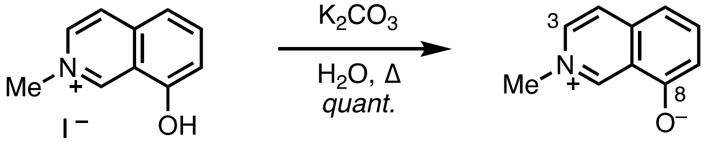

S6 **S1**

Isoquinolinium salt **S6** (60 mg, 0.21 mmol, 1.0 eq.) was dissolved in H_2O (2 mL) and K_2CO_3 (43 mg, 0.31 mmol 1.5 eq.) was added and the reaction heated to 70 ºC for 10 min. the mix was cooled and extracted with $CHCl_3$ (3 x 5 mL). The pooled organic extracts was dried over K_2CO_3, filtered and concentrated to give the zwitterionic **S1** as a dark red solid (34 mg, 0.21 mmol, quantitative). s IR ν 3357, 1644, 1574, 1440, 1338, 1202 cm^{-1}; ^1H NMR (CD_3OD, 500 MHz) δ 9.27 (s, 1H), 7.90 (dd, J = 7.0, 1.5 Hz, 1H), 7.82 (d, J = 7.0 Hz, 1H), 7.77 (t, J = 8.0 Hz. 1H), 6.90 (d, J = 8.0 Hz, 1H), 6.81 (d, J = 8.0 Hz, 1H), 4.25 (s, 3H); ^{13}C NMR (CD_3OD, 125 MHz) δ 172.2, 145.9, 140.2, 137.9, 132.1, 123.7, 122.6, 117.3, 107.6, 45.6; HRESITOFMS *m/z* 160.0755 $[M+H]^+$ (calcd. for $C_{10}H_{10}$ NO, 160.0757).

Table S3. ^1H NMR data for compounds **S1** and **S6** (CD_3OD, 500 MHz).

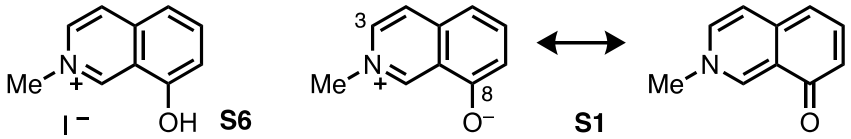

atom	**S6** ^{13}C	**6** ^1H	**S1** ^{13}C	**S1** ^1H
1	147.7	9.85 (s, 1H)	145.9	9.27 (s, 1H)
3	140.3	8.42 (d, *J* = 8.0 Hz, 1H)	140.2	7.90 (dd, *J* = 7.0, 1.5 Hz, 1H)
4	126.3	8.28 (d, *J* = 8.0 Hz, 1H)	123.7	7.82 (d, *J* = 7.0 Hz, 1H)
4a	139.2	-	137.9	-
5	120.7	7.66 (d, *J* = 10.0 Hz, 1H)	122.6	6.90 (d, *J* = 8.0 Hz, 1H)
6	136.3	8.05 (t, *J* = 10.0 Hz, 1H)	132.1	7.77 (t, *J* = 8.0 Hz. 1H)
7	114.4	7.27 (d, *J* = 10.0 Hz, 1H)	107.6	6.81 (d, *J* = 8.0 Hz, 1H)
8	158.7	-	172.2	-
8a	118.4	-	117.3	-
N-CH_3	~49.0	4.49 (s, 3H)	45.6	4.25 (s, 3H)
HO	–	[2]	–	

[2]Not observed
